# Mr. Wastell's Case of Thoracic Suppuration

**Published:** 1803-05-01

**Authors:** Henry Wastell

**Affiliations:** Member of the Royal College of Surgeons, London. Wellclose Square


### *To the Editors of the Medical and Physical Journal.*

GENTLEMEN,

YOU well know that all medical science at last centres in the cure of diseases, and that is the most useful part which enables us to effect it.

Although no *uniform* method of treatment is capable of effecting a cure in *all* the cases of internal suppurations of the chest, as difference of constitution, temperament, &c. has peculiar influence on the event; yet, I hope the following case of empyema, which occurred in May 1793, under the care of Dr. Saunders and myself, and which was inserted in the 5th Vol. of Memoirs of the Medical Society of London, (p. 215) may, when given more at large, be of some use to the medical practitioner.

It is perhaps as singular a complaint as is to be found in the records of medical history, and though it terminated successfully, yet we cannot be too cautious of entertaining hopes of a patient's perfect and permanent safety after such extensive and violent internal suppurations of the chest, until a sufficient time has elapsed. "Dr. Beddoes justly observes, in his late publication on the effects of Digitalis Purpurea, that in the reports of physicians concerning diseases of long continuance, it is a common fault that they drop the story too soon, leaving the intelligent reader uncertain, whether the long established habits of morbid action may not have returned after the apparent recovery."

It is now nine years since Mr. Metcalfe's illness, and I have the pleasure to enclose a letter which I received from him, dated at St. Petersburgh, June 19, 1802, which confirms his cure being permanent.

I also inclose a letter from my late worthy and intimate friend Mr. John Hunter, by which will be seen the small expectation we had of saving his life.

### THE CASE.

May 16, 1793, Mr. John Metcalfe, aged sixteen, of an athletic make, and sanguine habit, when in a violent perspiration from exercise, stood in his shirt exposed to a cold north wind. In the evening he complained of a sharp pain in his left breast, and across his loins; and, feeling chilly, he drank some warm brandy and water and went to bed. He was very feverish in the night, and rested badly, and in the morning of the 17th I was desired to see him.

He

He then complained of a pain across his chest, and particularly in the region of the left kidney; his skin was very hot, his face like scarlet, his breathing very difficult and painful; pulse 100, hard and full.

Eighteen ounces of blood were taken away, a large blister applied to the side, and the following medicines given:

℞. Nitr. purif. gr. xvj. cr. tartar gr. viij. sal ammon. gr. iv. sulph. aurat. ant. gr. ij. sumt. unam 2do. vel 3tia. quaq. hora ex paulo aq. hordeat. superbibendo cochl. iij. misturæ sequentis.

℞. Aq. puræ ℥iß. acet. scillit. mellis brittan aa. ℥j. M. ut supra exhibend.

18th. This morning his breathing easier, pains less, pulse 96, not so hard; had a motion in the night, made water with difficulty, and urine muddy.

℞. Aq. menth. sativ. ℥v. tinct. stomach. ʒv. acet. scillit. ℥j. sal. diuret. ʒij. syr. sacchar. ʒiij. M. sumat. cochl. iij. 2do. quaq. hora vel urgente dyspnœa.

In the evening very restless, pulse unequal and tense, skin very dry, and cough hard. Twelve more ounces of blood taken away; had two motions.

℞. Pulv. Doveri gr. iij. Kermes mineralis gr. ij. confect. cardiac q. s. fiat bolus 4tis hora sumend.

℞. Decoct. rad. senekæ ℥iß. spir. minderer. ʒiij. spir. nuc. mosch. ʒj. pulv. tragac. comp. ℈j. nitri. purif. ℈ß syr. sacchar ʒj. M. post. sing. bol. sumend.

19th. He was better; pulse 84, soft, skin moist, pain across his loins entirely gone, breathed easier, urine high coloured. Continued the same medicine, with lac. amygd. pro potu.

20th. This morning his breathing became very difficult, and the pain in his chest more severe; his pulse 110, and tense. Took away five ounces of blood, which, on standing, had a greenish film on top, and cupped. A laxative glyster was given.

Dr. Saunders was called in, who ordered eight ounces more blood to be taken away, and four grains of James's powder to be taken every four hours, with a saline draught. The blood had a yellow greenish crust; crassamentum of a loose texture.

25th. He continued the same medicines, with an occasional laxative of sal polycrest and rhubarb. Pulse 84; expectorated freely, breathed quick, cough frequent, urine natural colour, pain nearer to the vertebræ. Took three spoons full of a decoction of seneka root with nitre.

June 2d. Pain less severe, breathed better, skin moist,

 urine urine thick with red sediment. The left carotid artery beat 110 times in a minute; the pulse at the wrist 88.

4th. Pulse 86, weak; the pain shifted to the chest, his belly much swelled and very painful. Applied a blister to the part, and gave

℞. Pulv. scillæ. calomel aa. gr. j. extr. thebaic. gr. ß. M. f. pill. h. s. sumend. with a draught of almond milk and nitre.

5th. This pill generally procured a good night, and gave a stool with mucus in it. The pain shifted to the left shoulder.

12th. He continued much in the same way, the pain shifting from the shoulder to the *right* side, which was very acute when he coughed. Three ounces of blood were taken from his back by cupping, and his side was dry cupped, which gave great relief. Dr. Saunders was again consulted, who ordered,

℞. Sal c.c. gr. viij. confect. democrat. ℈j. aq. puræ ℥ij. spir. nitrid. ʒj. M. to be taken three times a day.

June 16th. Great tightness across the abdomen; pain in his side and cough the same, a laxative of rhubarb and sal polycrest was given; in good spirits.

17th. The pain got under the axilla and left shoulder.

22d. Pulse 100 and weak; great pain in his side; cannot rest on his right side; applied a blister, and continued his volatile anodyne draught every eight hours.

25th. Pulse 110 and weak; Dr. Saunders ordered P. Myrrh. gr. x. sal absynth. sal martis aa. gr. iv. spir. n. m. syr. balsam. aa. ʒj. M. bis die sumend. and volatile anodyne draught at bed time. His appetite good, and all sorts of fruit agreed with him, *except* strawberries, which always agreed before and after his illness.

July 3. A tumour appeared under his left nipple, attended with pain, and projection of his ribs; pulse 112, small. Dr. Saunders ordered emplastr. magnum gummos. parti dolenti. Chalybeate and anodyne draught continued as before.

14th. Pulse 120, weak; cough frequent, with acute pain in his breast; could lie on either side. ℞. Aq. hordeat tep. ℥v. oxymel simpl. ℥iß. tinct. scillæ. gutt. xxx M. sumt. cochl. iij. 4to. quaq. hora.

15th. Tumour larger; had good appetite; two motions in twenty-four hours, with very great sediment in his urine. Medicines continued as before.

20th. Tumour more prominent, fresh gum plaster applied. The pulsation of the carotid artery was 140 times in in a minute, that at the wrist 110. This day, he said, he felt something give way in his breast, the pain then ceased. His urine for many days past, deposited a large branny sediment. Continued the vol. anodyne draught at bed-time.

21st. His cough very frequent, breathing short, tumour larger, pulse 120, hard, small, and weak. ℞. Aq. pulegii ℥v. lac. ammon. ℥ij. oxymel scillit. ʒiij. elix. paregor. ʒijß. vin. antimon. ʒij. M. sumt. cochl. j. urgente tussi vel dyspnœa. Had six poultaceous stools, with much pus and mucus; these copious evacuations made him very faint; the pulse softer and fuller, carotid artery beat very quick, great sediment in his urine. He passed a very good night, coughed seldom, and had very little pain.

23d. I this day observed his heart to beat on the *right* side. Mr. Blicke, surgeon of St. Bartholomew's Hospital, met me this day to examine his side. The tumour was less, pulse slower and fuller, and the pain scarce perceptible; he could lie on his back and on either side; had three stools with much green mucus, made little urine. I substituted sal diureticus, gr. xv. with an opiate, instead of the volatile salt, at night, and a tea-spoon full of Castor oil next morning.

29th. Continued diuretic salt with opiate at night, ol. ricini. next morning in small quantities. Had no cough nor pain for some days past, until this day, when he drank porter, after which he expectorated much phlegm; had three solid stools with much mucus, and great sediment in urine; pulse 112, firmer, his heart still beating on the right side of the sternum.

31st. Medicines continued as before. Pulse 110, small and weak; urine clear, with a cloud near the bottom.

August 2, Finding himself better, went on-board his brother's ship; returned without pain or cough; pulse 100, soft, full, and firm; urine clear with a cloud, and mucus in his stools.

4th. Continued anodyne diuretic draught twice a day; he thinks himself better and stronger; cough a little troublesome; pulse 100, great sediment in his urine, had three stools but no mucus with them.

6th. A small tumour was perceived betwixt the seventh and eighth rib on the *right* side, the heart was still more on the same side; pulse 100, soft.

10th. Mr. Turnbull, surgeon to the Eastern Dispensary, examined him with me. When placed in a recumbent posture, the small tumour on the right side emptied itself;

 when when pressed upwards it gave great pain. The pulsations at the wrist 110, those of the heart more frequent.

12th. The rising of the ribs of the left side continued nearly the same; applied a fresh gum plaster to it; the small tumour betwixt the ribs on the right side became painful.

13th. Wrote to my worthy friend, Mr. John Hunter, to have his opinion; he was so employed with army business, he could not meet me, as you will see by his letter which I enclose.

14th. This morning his pulse was only 80 beats in a minute, soft and full. In the evening I was sent for, on account of a rising of phlegm in his throat with constant cough. On applying my hand to the tumour on the right side, I could perceive it fill and empty every time the patient coughed. Pulse very quick; gave small but repeated doses of a solution of vitriol. alb. which relieved him for the present.

16th. The tumour was larger, and filled at every expiration. Seeing the urgent necessity of an immediate opening from my own experience as well as the opinion of many eminent surgeons,* I proposed opening it, but was not permitted.

19th. The tumour on the right side was very painful, and discoloured, expectoration viscid, very great sediment in his urine, pulse unequal.

20th. This day at noon he had an incessant cough, and began to spit thick matter of a greenish colour; when I got to him at five o'clock, he had spit three half-pint basons full, and was like to be suffocated. As I went I met with Dr. Turnbull, Physician to the Eastern Dispensary, who accompanied me, and agreed that an *immediate* operation was necessary, which I performed by making an incision betwixt the seventh and eighth ribs on the right side, rather larger than two inches, and *nearer* to the *superior* edge of the lower rib, than the *inferior* edge of the upper, to avoid the risk of wounding the intercostal artery. Fifty-two ounces of thick matter were evacuated, similar to what he had spit up. Applied a poultice with flannel bandage and scapulary, and the patient was put to bed. The pulsation of the heart was near to the right axilla, and too

* Vide Warner's Cases in Surgery, p. 160, &c. Gooch's Cases, vol. ii. p. 139. Kirkland's Enquiry, vol. ii. p. 176, 179, &c. Duncan's Comment, vol. ii. p. 427. Dr. Hunter's Lectures. Mr. Bell's Medical Memoirs, vol. iii. p. 132.

too quick to be counted; the pulse at the wrist 136. His breathing, forty-eight times in a minute. He was placed on his right side, and gave volatile anodyne draught.

21st. He passed a very good night; a very great discharge from the wound; pulse 96, soft and full, with a florid colour in his cheeks. Dr. Saunders, his physician, and Mr. Haighton (now Dr. Haighton) came to see him. In the evening, pulse at wrist 120, small and weak. He coughed very little; lived on barley water and milk, had a good appetite, which never once failed him during his illness, but his strength much reduced. Volatile anodyne at bed-time.

22d. He passed a very good night; pulse 98, soft, full, and firm; a very great discharge on dressing the wound; no pain of his side, but a little soreness about the incision. Takes milk for breakfast and supper, wine and porter with his victuals at dinner. As the heart, notwithstanding the great evacuation of matter, still remained near the axilla, I wrote to Mr. John Hunter for his opinion, which he gave me in the letter I sent.

25th. The discharge from his side diminishing, and tumour under the left nipple much less; pulse at wrist 90, right carotid artery 130; his cough was much better, but very much reduced; the heart approaching nearer the left side ℞. Sal diuret. gr. xv. rubigo ferri, gr. v. confect. opiat. ℈j. Aq. pur. ℥iſs. spir. juniper comp. ʒij. M. h. s. sumend. omni nocte.

29th. Tumour on his left breast quite disappeared, and the heart *under* the sternum; it remained in this situation three days. His appetite good, in good spirits, could walk up stairs without his breathing being affected. Continued the same medicines.

September 1, He spit freely at night, but not in the day; as the discharge from the wound diminished, and a fullness appeared below the orifice, enlarged the aperture, and applied a poultice; pulse 98, fuller; had a good night.

4th. This is the first time the heart was felt on the LEFT side of the sternum; no pain, less expectoration, pulse 90, soft, full, and firm; breathes quite easy; went out in a coach for the last three days, and was much stronger.

6th. As the discharge from the side was much diminished, and spit near three ounces of pus every night, though none in the day time, from the experience of five successful cases of a scrophulous nature, under my care, I made two issues betwixt his shoulders, by means of caustic, to hold three peas each, and gave him tonics and volatile

 anodyne anodyne draught every night at bed-time. ℞. Pulv. myrrh. ʒj. sal absynth. sal martis aa. gr. xv. sacch. alb. ʒij. aq. puræ ℥viſs. spir. cinnamon ℥ſs. M. sumt. cochl. iij. bis de die.

6th. Pulse 92, soft and firm; the heart two inches on the *left* side of the sternum. His diet was milk and fruit, breakfast and supper; bread pudding and light animal food for dinner, with porter and wine; oysters when hungry; went out in a coach every day; sleeps well; generally three stools a day; urine natural.

15th. As he slept well, expectoration trifling, he left off the night draught, and continued the chalybeate mixture, with daily exercise.

19th. He gets stronger daily; breathes short; no expectoration when he coughs, which is but seldom; air passes through the wound in his side; little discharge from the wound, but much from the issues. The dressing to the wound was cerate saturnine with one-third cerate citrinum.

26th. He walked from Burr Street to Broad Street, distant a mile and a half, to see his physician, to let him know his heart was returned to its proper place, and walked back without the least inconvenience. He now gained strength rapidly, and began to recover his flesh and healthy florid complexion.

30th. He went to Stockton his native place by sea, taking with him some chalybeate pills and other medicines, and arrived safe much improved in his health.

October 16, Received a letter from him at Husworth, near Darlington, acquainting me he rode out every morning without fatigue, that the wound in his side was healed, but the issues continued to discharge freely. He continued to take two of his chalybeate pills every day; lived on milk morning and night, and light animal food with a few glasses of wine and ale after his dinner. He continued in the country, and finding himself in the most perfect health, he suffered the issues to heal in January, 1794. He came to London; and in October went to Elbing, in Ducal Prussia, and remained there and at Petersburgh, in the most perfect state of health, as appears by his letter I received, dated June 19, 1802.

To some persons, so minute a recital of facts may appear tedious and unnecessary; but they ought to consider that this was the first, and is still the most certain method of improving medicine. An account of so uncommon a disease, requires a circumstantial detail of all the circumstances, preceding, attending, and following the method of of treatment, in order to elucidate, if possible, the nature of the case. The most similar and successful case, which I know that resembles my patient's, is given by Dr. Farquharson, of Edinburgh, in the third volume of the Memoirs of the Medical Society of London, of a young gentleman, eight years of age, whose heart beat on the *right* side of the chest, in consequence of a considerable swelling on the *left* side of his breast. Two months after his first seizure, an opening was made into the cavity of the thorax through the most depending part of the tumour; though near two pounds of pus was discharged, yet it did not afford permanent relief until a long leaden canula was introduced, by the advice of Mr. Bell, which reached to the bottom of the cyst.

It may be useful to consider whether Mr. Metcalfe's case originated from constitution or temperament.

He says, in a note I received from him, in 1794, that about six years before, when about ten years of age, he had an abscess in his right side, and was under the care of the late Mr. Rudd, of Darlington, who opened it, and kept it open for three years; after it was healed, he had small abscesses on his left arm and clavicle, which soon got well by bathing it with Croft-Well water. From that period he enjoyed a good state of health until his last illness, and always lived in the most temperate manner.

The patient's father informs me, four of his children died of similar complaints. He has now living five sons and three daughters, all healthy men and women.

Does not this case afford a strong proof of the benefit of caustics and issues, whether the operation is performed or not?

I have had several opportunities of cases under my care, of their salutary effects in diseases of the chest and spine. See a case of a navy surgeon, in the Medical and Physical Journal, Vol. iv. No. 50; and also the good effects of caustics, in Dr. Beddoes's Observations on the Powers of Digitalis Purpurea, Appendix, No. 3, p. 101, 104, 109.

I am, &c.

HENRY WASTELL,

Member of the Royal College of Surgeons, London.

*Wellclose Square, April* 11, 1803.

