# Errata

**Published:** 1803-07-01

**Authors:** 


### ERRATA.

Vol. ix. Page 427, line 12, *read* ℥vifs. *vice* ℥ifs.

432, 19 from bottom, *read* Hurworth, *vice* Husworth.

433, 6 ditto, *read* Vol. ix. *vice* iv.

